# From ice to bugs: polymers and sugars to address healthcare challenges

**DOI:** 10.4155/fsoa-2016-0043

**Published:** 2016-06-28

**Authors:** Matthew I Gibson

**Affiliations:** 1 Department of Chemistry & Warwick Medical School, University of Warwick, Gibbet Hill Road, Coventry, CV4 7AL, UK

**Keywords:** biosensor, cryopreservation, glycans, infection, multivalancy, pathogen, polymers

## Abstract

**Matthew I Gibson talks to Francesca Lake, Managing Editor:** After completing his PhD at the University of Durham (UK), Matthew moved to the Ecole Polytechnique Federale de Lausanne (Switzerland) where he researched nanoparticle delivery technology. In 2009, he then moved to the University of Warwick (UK), to establish his independent research group. He has since been promoted to assistant (2012), associate (2015) and full (2016) professor. His interdisciplinary group undertakes research with the core theme of using biomaterials to improve health. This spans infection to regenerative medicine, and to fundamental polymer chemistry.

## Q Can you tell us about what led you to where you are today?

I am not entirely sure when I decided I wanted to be an academic, but probably during my final year research project. This was on polymer synthesis and I was encouraged to do a PhD by my supervisors (Professor Lian Hutchings and Professor Randall Richards). My PhD was really good fun; the environment was really friendly and lots of opportunities to not just do science; my supervisor (Professor Neil Cameron, who is now a colleague at Warwick!) encouraged us to attend a lot of large conferences. When I attend conferences I always try to attend talks I know nothing about, which I find really stimulate new ideas and ‘out-of-the-box’ solutions.

The motivation for our research is that in the past century advances in food supply, water purity and antibiotics have dramatically improved human health. Now we are in a situation where we have an aging population and emergence of antibiotic resistance, so we need to find new solutions to the problems borne from our previous successes. I am motivated to use ‘non-traditional’ solutions to this, which typically involve polymers and carbohydrates – the most exciting and versatile two classes of molecules (in my biased opinion)!

## Q How do you go about determining which medical issues might benefit from your approach?

This is an excellent question. Quite often, as chemists, we produce a lot of answers (e.g., molecules/analytics), for which we go in search of questions we can hopefully apply it to. We often do this, as fundamental discoveries from one discipline may have unknown benefits in new ones. However, I have always been keen on trying to understand the medical or biotechnological problems and then work toward the solution. This was the rationale behind me being the first joint appointment between our Chemistry Department and Warwick Medical School. Rather than having an office in the Medical School, I try to meet with colleagues to discuss what they are working on and see how either our chemical tools can help them, or (and arguably more importantly) put them in touch with my other colleagues to build the teams to address big problems. A recent example of this was a series of chance conversations with Nick Waterfield (Medical School) [1], who studies pathogens, especially insect ones. After a few meetings we realized he had identified a series of unique chemical structures that would help our work; within a year we have funding for two PhD students and a postdoc and are now considering a spinout company. My early work on cryopreservation was enabled by Daniel Mitchel (Medical School immunologist) and a former colleague Manu Vatish (now at Oxford, a gynecologist) who arranged access to the cells laboratories and provided encouragement and support, and we continue to publish together on this [2–4].

## Q What do you find most rewarding about your work?

The most rewarding part is definitely seeing talented students and postdocs coming into the laboratory and developing. Former students of my group have embarked on academic, industrial and teaching careers and hopefully the experience in the research laboratory has helped them. Also, being recognized by your peers is also very rewarding – less than 10 years ago, I was still a PhD student and now these people, whose work inspired me, I can now call friends and colleagues. I still find it exciting to get a grant or paper accepted; knowing that others saw the interest and potential of our work is really satisfying.

## Q What do you find most challenging about your work?

Well, all academics will answer that we need more funding and less paperwork!

But seriously, balancing your commitments is a challenge; as an academic we essentially lead a small business (sourcing grants, employing people) but also work in a large, complex organization that has to balance the needs of very diverse departments. We have to not just raise funds, but deliver on it, and in parallel teach the next generation, do outreach and try to translate fundamental research findings into application. Being an academic we have to juggle many different hats, which is actually fun, but at times challenging.

## Q You recently presented work on a new ‘Chemical Tongue’ approach to biosensing – can you tell us a little about this concept?

Carbohydrate (sugars) are crucial signaling molecules in our body, and they coat the surface of our cells. Many pathogens have evolved to exploit these sugars, so that they stick to them as the first stage of infection. We are working on a sensing approach whereby we mimic the surface of a cell on a nanoparticle (or other surface) for which when the pathogen binds, we generate a signal. We have particularly used this for cholera toxin detection but are moving to other pathogens. This approach works, but we need additional specificity – you can achieve this by either using more complex sugars, which are hard to make or expensive to buy. So we instead make ‘multiplexed’ sensors where we have a series of inputs (sugars) that we train to the pathogen. This works like a tongue – we only have five different tastes, but we are trained to use these to identify foods. We do the same, with a few sugars to identify pathogens [5–7].

## Q What is next for this research?

We have shown it works in principle but we really need to translate it into something closer to a real device for analytics, and also apply it in complex media – by this we mean urine, blood or dirty water. A point-of-care device that needs lots of processing steps is not really a good solution. We are working toward this, and have some new data showing that we can overcome these challenges – keep an eye out for new papers!

## Q You also work on developing materials to enhance cryopreservation – how is that research progressing?

This is really progressing fast and was the first area of research in my independent group. [2–4] We are interested in a protein called an antifreeze glycoprotein (AFGP). These are made by fish in the Arctic to help them survive low temperatures. AFGPs are remarkably potent inhibitors of ice growth (i.e., what happens to old ice cream in your freezer) and this is a problem in cryopreservation of cells – ice crystals grow and kill the cells. We wanted to make synthetic polymers that can mimic AFGPs and use these to help us freeze donor cells for regenerative medicine. So far we have identified new polymers [8,9] that mimic AFGPs but are far cheaper and easier to make and have shown that we can use these to enhance the cryopreservation of blood, but also primary (fresh) liver cells.

## Q What implications could this have in healthcare?

We think this will have a big impact. The main challenge of cryopreservation is actually the additives used – typically large amounts of organic solvents (antifreeze) are added to control the ice growth. This works well in some cases, but removing the solvents is hard and the cells do not really like the high concentrations, which can lead to toxicity. If we could remove the need for solvents and just use low concentrations of our polymers, we could potentially freeze more and different cell types and make the process a lot easier.

When you consider the huge advances in stem cell biology and regenerative medicine (e.g., for growing new tissue) the next obvious step is the logistical challenge of getting the cells/tissue from laboratory to patient, which will need something like cryopreservation to let them be stored, banked, transported and thawed (in much the same manner as frozen food distribution works).

## Q What else are you researching at the moment?

We use a lot of polymer science in my laboratory, so we have several projects on synthetic polymer chemistry, characterization and applications. Particularly in the design of polymers that can respond to biochemical gradients to give a response – this might be a color change, or to trigger cell uptake, for example. We also work a lot with colleagues in the School of Life Sciences and the Medical School and are looking at new drug candidates and delivery systems.

## Q Glycomaterials have been called a ‘hot topic’ for the next few years. What other research is happening in this area at the moment that is exciting you?

Glycoscience is always limited by the availability of the proteins and the sugars. Advances in biotechnology is really helping this – enzymatic synthesis is enabling easier access to complex sugars and I can foresee huge advances in this in the coming years. Another emerging area is cryo-electron microscopy. The resolution of electron microscopy is really increasing and being able to get structural information about proteins, without crystallizing them (which is hard, and we would not even attempt to do in my laboratory), will revolutionize our ability to understand how glycans/proteins interact.

The combination of accelerated and scalable synthesis using material chemistry tools such as what we are developing with the new biotechnology and analytics should really push glycoscience forward and enable us to translate it from laboratory to clinic/industry.

## Q Are there any other medical applications you can particularly see glycoscience helping us with?

This is clear to me; infection. Glycans are crucial in immunology, pathogen binding and signal transduction and if we can understand and mimic these we can make huge advances. For example, synthetic glycans have been developed as vaccines (with their structure designed to stimulate the immune system). With new and emerging threats like Zika and Ebola, as well as the re-emergence of ancient diseases such as tuberculosis, glycoscience has never been more important. The development of anti-infectives, where rather than killing pathogens, they are just stopped from adhering to the host, is also a hot area, where glycan mimetics could make a huge impact in the near future.

## Q Finally, if you had unlimited resources available, what research would you perform & why?

Relating to our cryopreservation work, I would really want to push to understand the ice/water interface and how antifreeze proteins affect it. Despite water being fundamental to life, we still have many questions about it. For example, ice is one of the few solids that has lower density than the liquid and the process of nucleation is not understood. Using modern high-resolution analytics, we could really probe this complex interface, which would have huge implications in a range of fields from cryopreservation, to self-de-icing cars, and even to space exploration, where ice formation is a huge problem.

## References

[1] Warwick Medical School http://www2.warwick.ac.uk/fac/med/staff/nwaterfield/.

[2] Deller RC, Vatish M, Mitchell DA, Gibson MI (2014). Synthetic polymers enable non-vitreous cellular cryopreservation by reducing ice crystal growth during thawing. *Nat. Comm.*.

[3] Deller RC, Vatish M, Mitchel D, Gibson MI (2015). Glycerol free cryopreservation of red blood cells enabled by ice recrystallization inhibiting polymers *ACS*. *Biomater. Sci. Eng.*.

[4] Deller RC, Mitchell DA, Vatish M, Pesin J, Gibson MI (2016). Enhanced non-vitreous cryopreservation of immortalized and primary cells by ice-growth inhibiting polymers. *Biomater. Sci.*.

[5] Richards SJ, Otten LC, Gibson MI (2016). Glycosylated gold nanoparticle libraries for label-free multiplexed lectin biosensing. *J. Mater. Chem. B.*.

[6] Otten LC, Gibson MI (2015). Discrimination between lectins with similar specificities by ratiometric profiling of binding to glycosylated surfaces; a chemical ‘tongue’ approach. *RSC Adv.*.

[7] Otten LC, Vlachou D, Richards SJ, Gibson MI (2016). Glycan heterogeneity on gold nanoparticles increases lectin discrimination capacity in label-free multiplexed bioassays. *Analyst*.

[8] Congdon TC, Notman R, Gibson MI (2013). Antifreeze (glyco)protein mimetic behavior of poly(vinyl alcohol): detailed structure ice recrystallization inhibition activity study. *Biomacromolecules*.

[9] Mitchell DE, Cameron NR, Gibson MI (2015). Rational, yet simple, design and synthesis of an antifreeze-protein inspired polymer for cellular cryopreservation. *Chem. Commun.*.

